# Investigate of *LOC101928988* Regulatory Effect on the *DAPK2* Transcription in Breast Tumors

**DOI:** 10.1002/cnr2.70115

**Published:** 2025-02-19

**Authors:** Mohammadreza Saberiyan, Nazila Ghasemi, Negar Jafari, Mahboubeh Sadeghi, Abbas Ghaderi, Pegah Mousavi

**Affiliations:** ^1^ Student Research Committee Hormozgan University of Medical Sciences Bandar Abbas Iran; ^2^ Department of Medical Genetics, Faculty of Medicine Hormozgan University of Medical Sciences Bandar Abbas Iran; ^3^ Department of Biology, Jahrom Branch Islamic Azad University Jahrom Iran; ^4^ Department of Cardiology, School of Medicine Urmia University of Medical Sciences Urmia Iran; ^5^ Shiraz Institute for Cancer Research, School of Medicine Shiraz University of Medical Sciences Shiraz Iran; ^6^ Department of Immunology, School of Medicine Shiraz University of Medical Sciences Shiraz Iran; ^7^ Molecular Medicine Research Center Hormozgan Health Institute, Hormozgan University of Medical Sciences Bandar Abbas Iran

**Keywords:** apoptosis, autophagy, breast cancer, *DAPK2*, *LOC101928988*

## Abstract

**Background:**

The World Health Organization has mentioned breast cancer holds the highest incidence rate among all types of cancer globally. Death‐associated protein kinase 2 (*DAPK2*) is a serine/threonine kinase linked to various forms of malignancy, such as breast cancer. This protein assumes a pivotal function in a multitude of cellular mechanisms, including apoptosis, autophagy, and cell migration.

**Aims:**

This study aimed to study the *LOC101928988* regulatory effect on the *DAPK2* expression in breast cancer.

**Methods:**

In this study, 38 paired tumoral and normal tissues were selected from patients. Quantitative real‐time PCR was used to analyze the expression of *DAPK2* and *LOC101928988*. The interactions of *DAPK2* and its intermediate elements with *LOC101928988* were predicted by docking analysis.

**Results:**

The expression of *DAPK2* and *LOC101928988* was downregulated in tumor tissues compared to the control group. Further analysis revealed a significant positive correlation between *DAPK2* and *LOC101928988* levels in tumoral and adjacent normal tissues. A comparison of gene expression between different grades, stages, and HER2 statuses showed significant findings. ROC curve analysis of *DAPK2* and *LOC101928988* expression revealed 77% and 72% AUC for BC tissue, respectively.

**Conclusions:**

Overall, our results suggest that alterations in the levels of *DAPK2* and *LOC101928988* may be involved in tumor initiation and progression in breast cancer. It has also been reported that *LOC101928988* probably has a role in regulating *DAPK2* expression through interaction with transcription factors.

**Abbreviations**
AUC: area under the curve
cDNA: complementary DNA
COREST: corepressor for element‐1‐silencing transcription factor
CTCF: CCCTC‐binding factor
*DAPK2*
: death‐associated protein kinase 2
ER receptor: estrogen receptor
EZ2H: enhancer of zeste homolog 2
HNRNPL: heterogeneous nuclear ribonucleoprotein L
lncRNAs: long non‐coding RNAs
ncRNAs: non‐coding RNAs
NSCLC: non‐small cell lung cancer
PR receptor: progesterone receptor
RBPs: RNA‐binding proteins
REST: RE1‐silencing transcription factor
ROC curve: receiver‐operating characteristic curve
TFs: transcription factors
WHO: World Health Organization

## 1 Introduction

The World Health Organization (WHO) is alarmed by breast cancer's highest incidence rate among all types of cancer among women worldwide. In 2023, ~297 790 newly detected cases of invasive breast cancer are expected in women residing in the United States [1]. Breast cancer is a complex disease and its exact causes are not fully understood [2]. Scientific investigations have indicated that modifications in genes implicated in apoptotic and autophagy mechanisms play pivotal roles in the progression and advancement of this condition [3, 4, 5]. Apoptosis is an innate process that aids in the elimination of damaged or aberrant cells from an organism, whereas autophagy functions as a mechanism for salvaging impaired or dysfunctional cellular constituents [6, 7, 8, 9, 10]. The malfunction of apoptotic and autophagy genes can result in the survival and proliferation of cancerous cells, ultimately culminating in the expansion and dissemination of tumors [11, 12, 13]. Several essential genes are involved in breast cancer apoptosis, including *TP53*, *BCL2*, and *BAX* [14, 15, 16]. In addition, autophagy genes, such as *BECN1*, *ATG5*, and *ATG7*, are dysregulated in breast cancer [17, 18]. Among the various genes involved in autophagy, death‐associated protein kinase 2 (*DAPK2*) has garnered increasing attention because of its regulatory role in both autophagy and apoptosis [19, 20].

*DAPK2* is a serine/threonine kinase that has been linked to various malignancies, such as breast cancer [21]. This protein assumes a pivotal function in many cellular mechanisms, including apoptosis, autophagy, and cell migration [22, 23, 24]. *DAPK2* actively participates in regulating the cytoskeleton, which is responsible for upholding the configuration and composition of cells [25]. Its role in tumor suppression through the modulation of these pathways has been increasingly acknowledged [26]. *DAPK2* not only promotes apoptosis through programmed cell death but also augments autophagic activity by interacting with essential proteins including SQSTM1/p62 and beclin‐1, hence affecting cellular homeostasis and survival during stress conditions [27]. *DAPK2* phosphorylates SQSTM1/p62 at serine 403, which enhances the binding of SQSTM1/p62 to LC3, a protein involved in autophagosome formation. This interaction promotes clearance of damaged or unwanted proteins by autophagy [28, 29]. Moreover, it was found that *DAPK2* plays a regulatory role in the mTORC1 pathway, which is a crucial modulator of autophagy [25]. *DAPK2* exerts its inhibitory effects on mTORC1 by phosphorylating and activating TSC2, a positive promoter of mTORC1 [30]. It is improbable that *DAPK2* possesses the capability to interact with beclin‐1, a factor that plays a crucial role in the inception of autophagy, as well as Bcl‐XL, a protein that exhibits anti‐apoptotic properties and can hinder autophagy [31]. Phosphorylation of serine 90 on beclin‐1 by *DAPK2* has been demonstrated to augments its interaction with Vps34, a kinase that is important in the initiation of autophagy. This particular phosphorylation event facilitates autophagy [32].

Understanding the functions of *DAPK2* is essential, as it reveals not only its roles in autophagy and cytoskeletal regulation but also its significant implications in cancer biology [33]. The research has shown that *DAPK2* is a critical factor in various malignancies, including lung, colorectal, and thyroid cancer. In the case of lung cancer, there is a connection between lower levels of *DAPK2* expression and heightened tumor proliferation, along with an unfavorable prognosis [26, 34]. Similarly, in colorectal cancer, reduced *DAPK2* expression has been associated with tumor advancement and the spread of cancer cells [35]. Moreover, *DAPK2* plays a crucial role in the initiation and progression of thyroid cancer, as it contributes to tumor development and the ability to resist TRAIL‐induced apoptosis through autophagic degradation of I‐κBα [36]. Despite its established roles, the potential of *DAPK2* as a therapeutic target in cancer treatment remains an active area of research, underscoring the complexity of its function in cellular processes [37]. Thus, further investigation of *DAPK2* is vital for understanding tumor biology and exploring new therapeutic strategies.

ncRNAs are various types of RNAs without protein‐coding functions, and regulation of gene expression has been identified as the most important function of them [38]. They are recognized as important regulators of genes involved in various physiological processes [39]. Long noncoding RNAs (lncRNAs) are a subtype of ncRNAs that are typically characterized as transcripts exceeding 200 nucleotides in length that do not undergo protein translation [40]. RNA‐binding proteins (RBPs) belong to a category of molecules that can bind to distinct RNA sequences and thereby contribute to various processes involving RNA, such as processing, transport, stability, and translation [41]. The interaction between RBPs and lncRNAs takes place through diverse mechanisms, governing the regulation of their stability and function, and ultimately, gene expression [42, 43]. A notable instance of this phenomenon is observed in the interaction between RBP HuR and lncRNA NEAT1, where HuR stabilizes NEAT1, consequently facilitating the formation of paraspeckles and leading to the alteration of gene expression [44]. This intricate interplay between RBPs and lncRNAs underscores the critical role of ncRNAs in fine‐tuning gene expression and cellular responses.

A lncRNA named *LOC101928988* with accession code NR120344.1 in NCBI was transcribed antisense from the *DAPK2* gene. The expression effects of this gene and lncRNA in breast cancer have not yet been investigated. The objective of this study was to examine the expression profile of *DAPK2* and its antisense lncRNA *LOC101928988* within the mTORC signaling pathway in breast cancer, focusing specifically on their involvement in autophagy. Our aim was to evaluate the potential impact of these molecules on the regulation of autophagy during the development and progression of breast cancer and to assess their viability as therapeutic targets. By analyzing the expression patterns in breast cancer tissues and their adjacent normal tissues, we aimed to gain a deeper understanding of the molecular mechanisms that cause dysregulation of autophagy in breast cancer and to discover new biomarkers for diagnosing and treating breast cancer patients.

## 2 Subjects, Materials, and Method

### 2.1 Ethics Statement

The Hormozgan University of Medical Sciences Ethics Committee authorized the study procedures (IR.HUMS.REC.1402.294), and all enrolled patients provided informed consent. Data related to clinicopathology were obtained from the patient records. This study was conducted in accordance with the Declaration of Helsinki (Association, 2019).

### 2.2 Sample Collection

A total of 38 tumor samples along with adjacent normal tissues were collected from patients who underwent breast surgery, with their consent, at the MRI hospital located in Shiraz, Iran. Fresh cancerous and adjacent non‐tumor specimens were rapidly frozen in liquid nitrogen and subsequently preserved at −80°C immediately after resection to facilitate RNA extraction. It should be noted that the patients had not received any previous treatment, and their carcinoma diagnosis was confirmed by pathologists situated within the hospital premises. The clinical data related to the specimens from the patients are given in Table 1.

**TABLE 1:** Clinical data of the patients with IDC.

Parameter	Cases (*n*)
Age (average)	
≤ 50	22
> 50	16
Stage	
Ι, II	29
III	9
Grade	
Ι, II	28
III	10
Tumor size	
< 2 cm	17
2–5 cm	19
Lymph node metastasis	
Yes	17
No	21

### 2.3 RNA Extraction and qRT‐PCR

Total RNA was extracted using a Total RNA Extraction Kit A101231 (Parstous, Iran). Reverse transcription was performed on RNA samples to produce complementary DNA (cDNA) using the Easy cDNA Synthesis Kit (Parstous, Iran). Finally, gene expression was quantified using real‐time PCR with SYBR Green Master Mix (Ampliqon, Denmark). *β‐Actin* served as an endogenous control. The Pfaffl method was used to normalize the gene expression levels. The primer sequences used are listed in Table 2.

**TABLE 2:** List of primer sequences.

Gene	Primer	Primer sequence
*β‐Actin*	F	5′‐GCC TTT GCC GAT CCG C‐3′
	R	5′‐GCC GTA GCC GTT GTC G‐3′
*DAPK2*	F	5′‐GACTTTGGTCTGGCTCACGA‐3′
	R	5′‐GATGACGCCTATGCTCCACA‐3′
*LOC101928988*	F	5′‐GAGTCCGAAGACCCAAGTTCA‐3′
	R	5′‐CTCTCCACCTTTTCCATTCGC‐3′

Abbreviations: F, forward; R, reverse primer.

### 2.4 Analysis of Molecular Docking

Many biological activities depend on interactions between nucleic acids and proteins. Understanding their structure facilitates the development of targeted treatments. Molecular docking is essential for identifying complex structures owing to its cost and complexity [45]. In this study, RNA‐protein interactions were predicted by docking analysis. The RNA Alifold web server (http://rna.tbi.univie.ac.at) predicted the secondary structure of lncRNAs. RNA Alifold, designed with user‐friendliness in mind, predicts an optimal secondary structure for a set of aligned sequences with maximum limits of 3000 nucleotides and 300 sequences [46]. Then, we obtained the 3D RNA structure from the RNA composer database, available at https://rnacomposer.cs.put.poznan.pl/, which employs a machine translation approach based on the RNA FRABASE database [47]. Next, we used SWISS‐MODEL (https://swissmodel.expasy.org/), a powerful program designed for automated protein homology modeling, to design the 3D structure of the protein in PDB format [48]. The HDOCK web server (https://hdock.phys.hust.edu.cn) was used for docking analysis [49].

### 2.5 Statistical Analysis

REST 2009 software was used for qPCR data analysis. In addition, Graph Pad/Prism 8.0.2 program was used for statistical analysis to compare gene expression levels among the groups under study. Data are presented as mean ± SD. Kruskal–Wallis and Mann–Whitney tests were employed to compare unpaired data after investigating the normality of data distribution using the Kolmogorov–Smirnov test. Spearman's correlation coefficient performed correlation analysis and *p* < 0.05 was considered a significance level.

## 3 Results

### 3.1 *DAPK2* Expression

**FIGURE 1:**
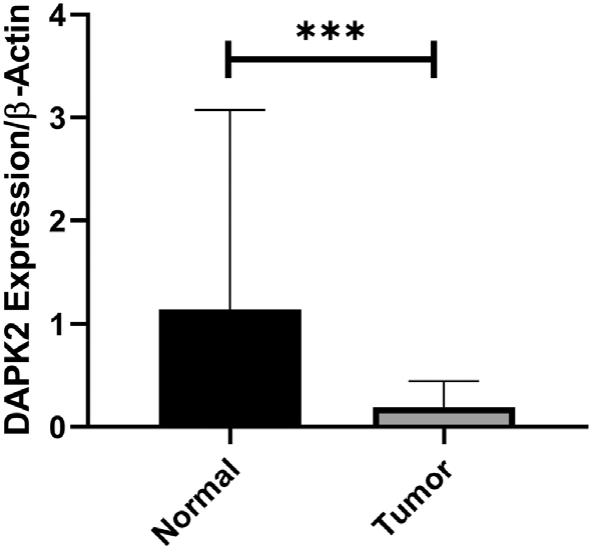
The expression levels of *DAPK2* in normal and tumor tissues. Tumoral samples showed a 5.24‐fold reduction in *DAPK2* expression (***p = 0.011).

**FIGURE 2:**
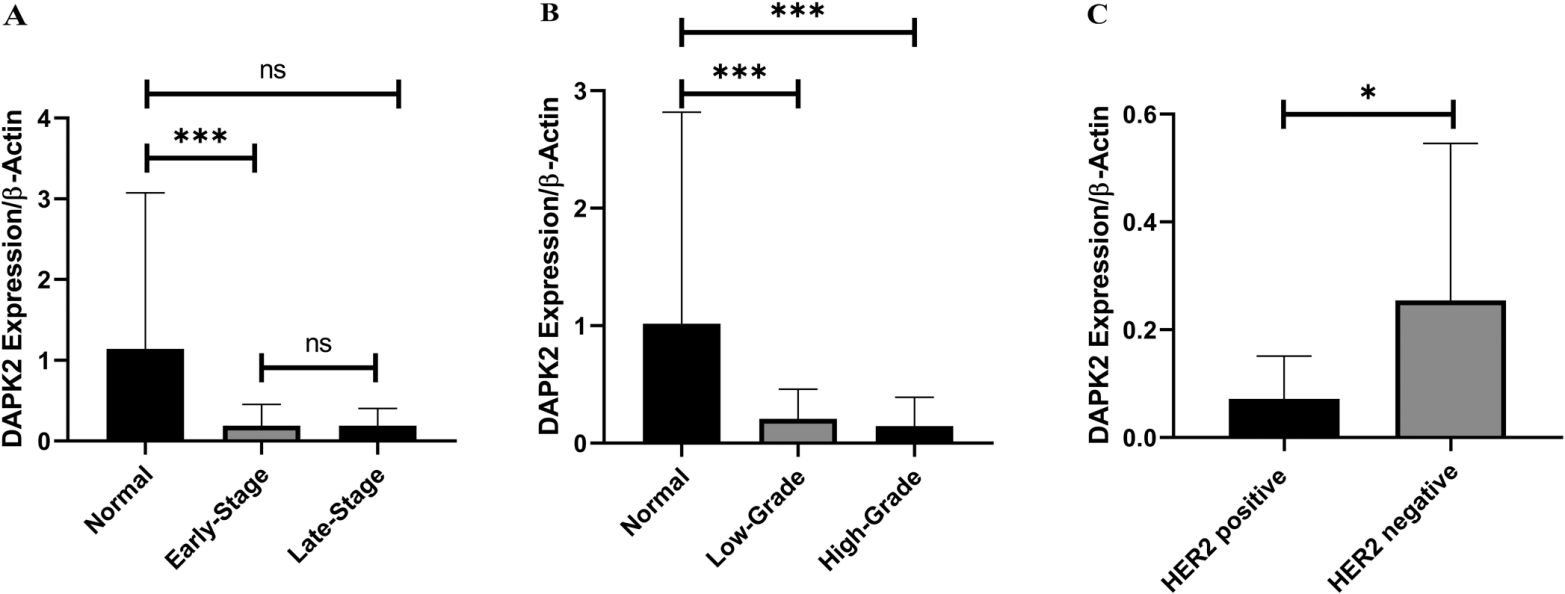
The expression of *DAPK2* in early‐stage and late‐stage tumors compared to normal. The data are represented as the mean ± standard deviation (SD). Statistical analysis was conducted using one‐way ANOVA with post hoc tests, and significance levels are indicated by asterisks (***p < 0.001, *p < 0.05, ns = not significant). (A): *DAPK2* expression in normal breast tissue compared to early‐stage and late‐stage breast cancer. The expression is significantly reduced in early‐stage cancer (***p < 0.001) and late‐stage cancer (***p < 0.001) compared to normal tissue, with no significant difference between early‐stage and late‐stage cancer (ns). (B): Comparison of DAPK2 expression between normal breast tissue and low‐grade and high‐grade breast cancer. Both low‐grade and high‐grade cancers show significantly lower expression levels than normal tissue (***p < 0.001), but there is no significant difference between the two cancer grades (ns). (C): *DAPK2* expression in HER2‐positive and HER2‐negative breast cancer. HER2‐negative samples exhibit significantly higher DAPK2 expression compared to HER2‐positive samples (*p < 0.05).

These findings indicated that *DAPK2* expression was markedly reduced in tumor samples compared to that in adjacent normal tissues (*p* < 0.05). Tumoral samples showed a 5.24‐fold reduction in *DAPK2* expression (*p* = 0.011) (Figure 1). It was also observed that *DAPK2* expression was significantly reduced in early‐stage tumors compared to adjacent normal tissues (*p* = 0.0003). However, in late‐stage tumors, the reduction in expression was borderline (*p* = 0.055). Moreover, *DAPK2* expression was significantly decreased in both low‐ and high‐grade tumors compared to adjacent normal tissues (*p* = 0.0016 and *p* = 0.0018, respectively). Notably, the expression of *DAPK2* was significantly lower in HER2^+^ tumors than in HER2^−^ tumors (*p* = 0.016) (Figure 2). Further analyses based on the status of ER and PR receptors, tumor size, lymph node involvement, Ki67 status, and family history did not reveal any significant relationship.

**FIGURE 3:**
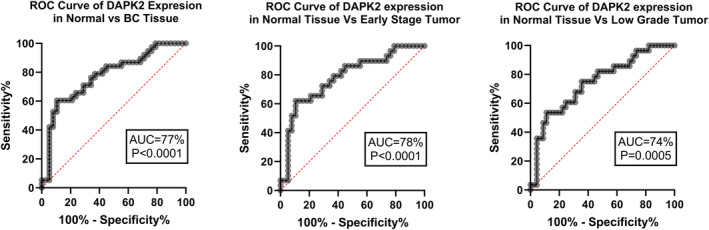
*DAPK2* expression ROC curve analysis revealed 77% AUC (*p* < 0.0001) for BC tissue and especially 78% AUC (*p* < 0.0001) for early‐stage tumors, and 74% AUC (*p* < 0.0001) for low‐grade tumors.

*DAPK2* expression ROC curve analysis revealed 77% AUC (*p* < 0.0001) for BC tissue, 78% AUC (*p* < 0.0001) for early‐stage tumors, and 74% AUC (*p* < 0.0001) for low‐grade tumors (Figure 3).

### 3.2 *LOC101928988* Expression

**FIGURE 4:**
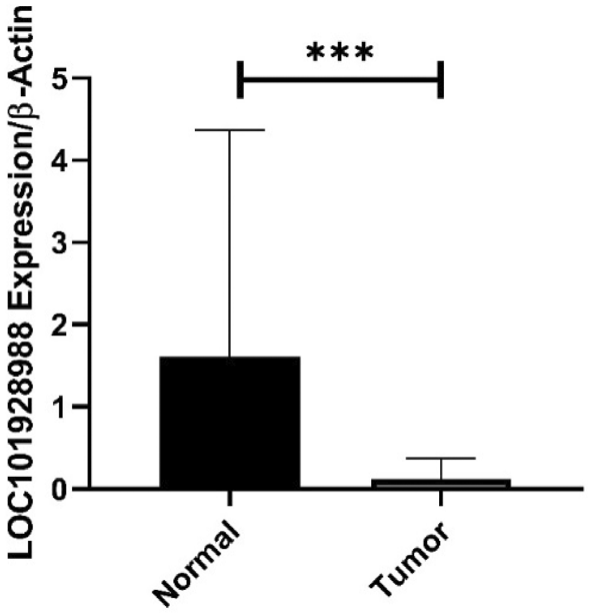
The expression of *LOC101928988* in normal and tumoral tissues. The data are presented as the mean ± standard deviation (SD). Statistical significance was determined using a two‐tailed *t*‐test, with significance levels denoted by asterisks (***p < 0.001). The expression of LOC101928988 is significantly higher in normal breast tissue compared to tumor tissue (*p* < 0.001).

**FIGURE 5:**
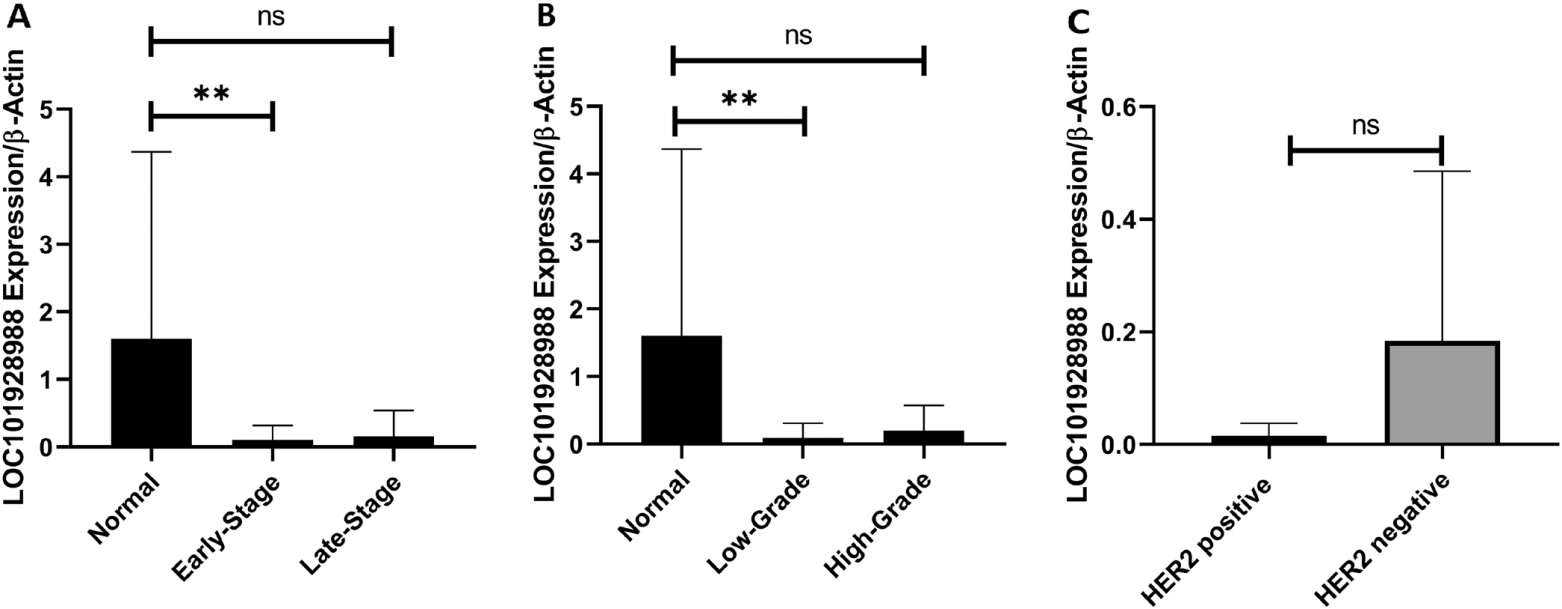
*LOC101928988* expression in normal early‐stage and late‐stage tumors. The data are expressed as the mean ± standard deviation (SD). Statistical analysis utilized one‐way ANOVA, accompanied by relevant post hoc tests, with significance levels shown by asterisks (**p < 0.01, ns = not significant). (A): The expression level in normal tissue is markedly elevated compared to early‐stage breast cancer (**p < 0.01) but exhibits no significant difference when compared with late‐stage breast cancer (ns). (B): A notable decrease in expression is evident in both cancer grades relative to normal tissue (**p < 0.01). (C): Expression of LOC101928988 in HER2‐positive and HER2‐negative breast cancer. No substantial difference is noted between the two groups (ns).

The expression of *LOC101928988* was downregulated in tumor samples compared to that in adjacent normal tissues. Tumoral tissues showed a 2.44‐fold increase in *LOC101928988* expression compared to the control group (*p* < 0.0001) (Figure 4). The expression of *LOC101928988* was significantly downregulated in early‐stage tumoral tissues in comparison to control group (*p* = 0.002), but no significant decrease was found in late‐stage tumoral tissues. Similarly, the expression of *LOC101928988* was remarkably downregulated in low‐grade tumoral tissues compared to that in the control group (*p* = 0.001), but no significant decrease was reported in expression in high‐grade tumoral tissues (Figure 5).

**FIGURE 6:**
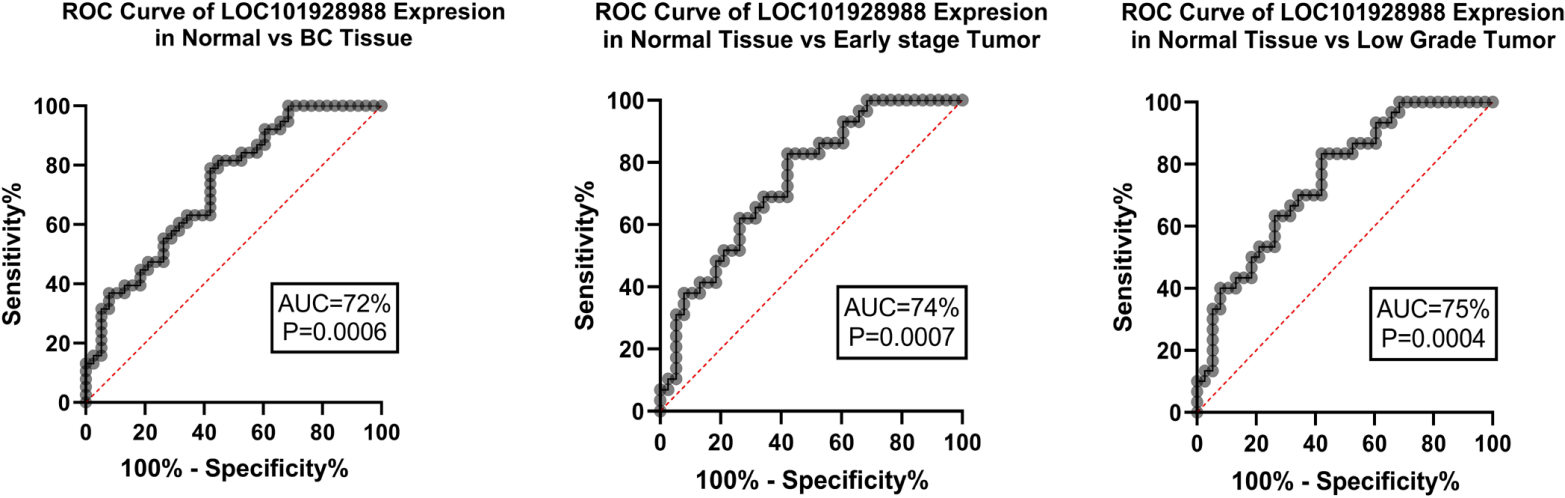
*LOC101928988* expression ROC curve analysis revealed 72% AUC (*p* = 0.0006) for BC tissue and especially 74% AUC (*p* = 0.0007) for early‐stage and 75% AUC (*p* = 0.0004) for low‐grade tumors.

*LOC101928988* expression ROC curve analysis revealed 72% AUC (*p* = 0.0006) for BC tissue, and especially 74% AUC (*p* = 0.0007) for early‐stage and 75% AUC (*p* = 0.0004) for low‐grade tumors (Figure 6). Further analyses based on the status of HER2, ER, and ki67 status, lymph node involvement, tumor size, and family history did not show any significant relationship.

**FIGURE 7:**
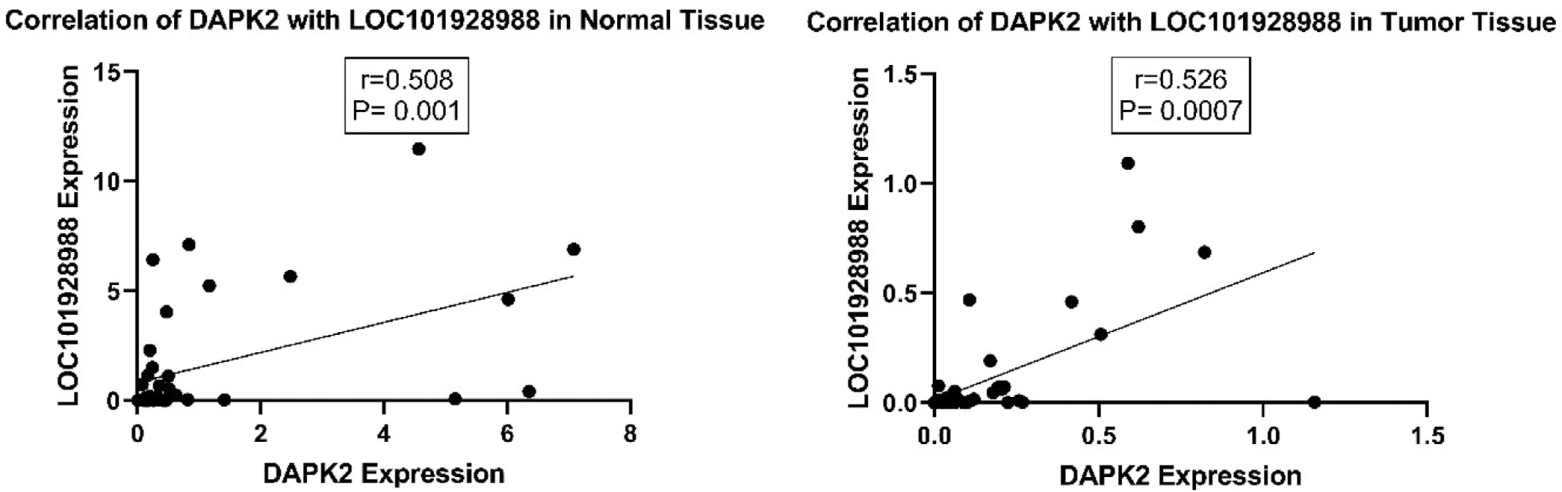
The scatter plots were created to display the correlations between *DAPK2* and the expression levels of the *LOC101928988* gene in tumoral and adjacent normal tissues. The positive correlation of *DAPK2* and *LOC101928988* (*R* = 0.508, *p* = 0.001) and (*R* = 0.526, *p* = 0.0007) in normal and tumor tissues, respectively.

Further analysis showed a meaningful positive correlation between *DAPK2* and *LOC101928988* expression levels in tumoral and adjacent normal tissues (Figure 7).

### 3.3 *LOC101928988* and Transcription Factors (TFs) Interactions

**FIGURE 8:**
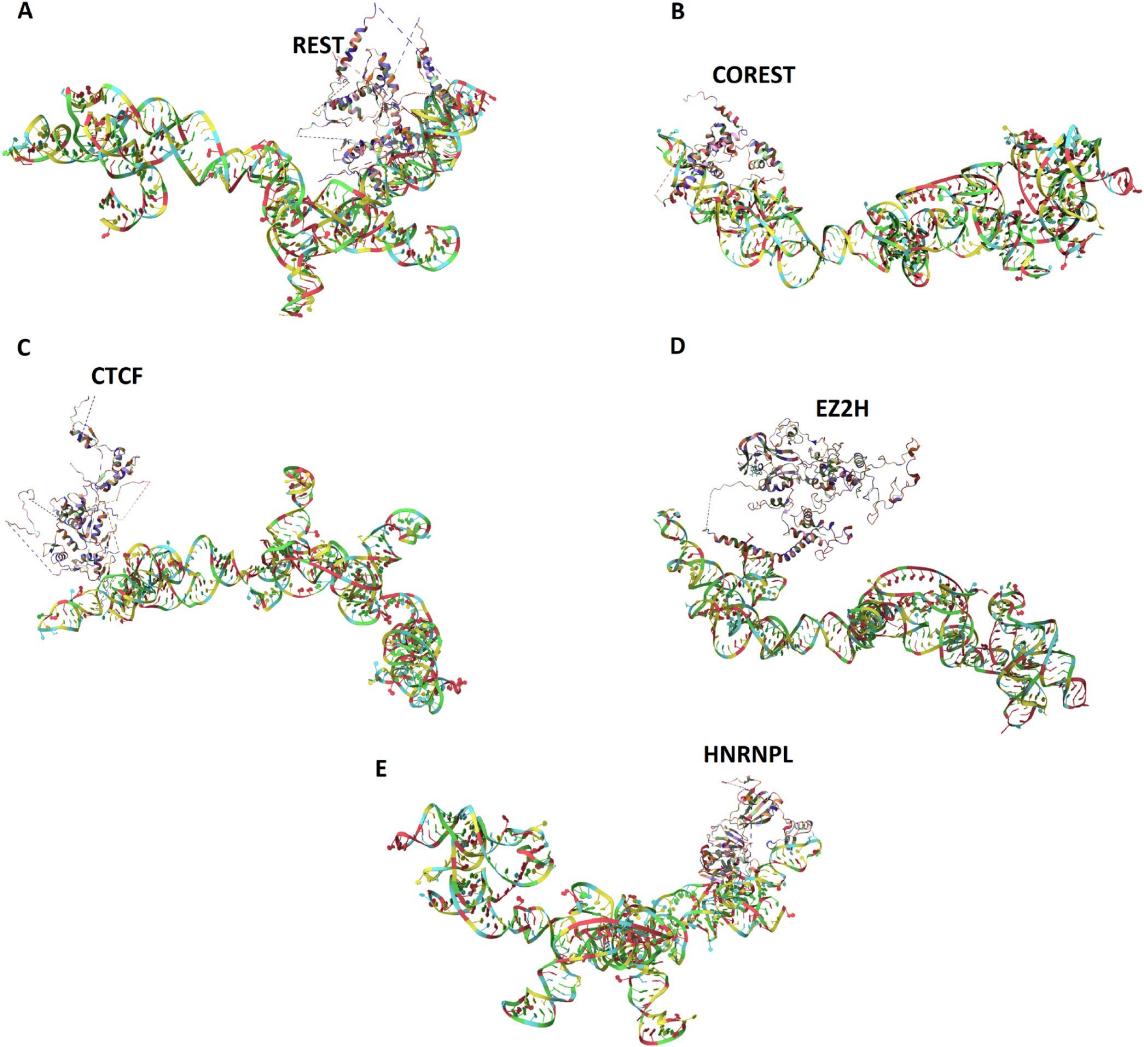
*LOC101928988* and transcription factors (TFs) interactions.

Docking analysis showed that *LOC101928988* could interact with, HNRNPL, CTCF, EZ2H, REST, and COREST that have binding sites in *DAPK2* Promoters (chromosome 15: 64044400–64 047 201), with a docking energy score of −408.05, −316.24, −373.84, −332.14, and −385.68, respectively (Figure 8).

## 4 Discussion

Genetic mutations, chromosomal instability, epigenetics, and ncRNAs are the major components contributing to tumor development in breast cancer [50, 51].

Our results revealed that the expression of *DAPK2* and *LOC101928988* was downregulated in tumoral tissues compared to that in the control group. Further analysis indicated a meaningful positive correlation between *DAPK2* and *LOC101928988* expression in tumoral and adjacent normal tissues. Our results revealed that the expression of DAPK2 and LOC101928988 was downregulated in tumoral tissues compared to that in the control group, indicating their potential roles in breast cancer pathogenesis. The significant positive correlation between DAPK2 and LOC101928988 expression levels suggests that these genes may interact in regulatory pathways critical for tumor development, which could be a therapeutic target. Furthermore, considering the multifactorial nature of breast cancer, strategies such as repurposing drugs and administering various vitamins, including vitamin E, as prophylactic measures may have a tumor‐modulatory effect with a positive impact on breast cancer outcomes [52].

The *DAPK2*'s expression level has been found to be considerably downregulated in early‐stage tumors compared to adjacent normal tissues. Moreover, the expression level of *DAPK2* was notably decreased in both low‐ and high‐grade tumors compared to that in adjacent normal tissues. HER2‐positive tumors exhibited significantly lower expression level of *DAPK2* than HER2‐negative tumors. Similarly, when compared with adjacent healthy tissues, the expression of *LOC101928988* was considerably lower in early‐stage tumor tissues. Low‐grade tumoral tissues also exhibited a significantly lower expression of *LOC101928988* than adjacent normal tissues. ROC curve analysis of DAPK2 expression revealed an AUC of 77% for BC tissues, with a particularly high AUC of 78% for early‐stage tumors and 74% for low‐grade tumors. In contrast, ROC curve analysis of *LOC101928988* expression revealed an AUC of 72% for BC tissues, with a particularly high AUC of 74% for early‐stage tumors and 75% for low‐grade tumors.

Research has shown that *DAPK2* plays a dual role in cellular processes, particularly autophagy and apoptosis [26]. Nevertheless, it is recognized to function as a tumor suppressor in various types of cancers, including breast, ovarian, colorectal, bladder, gastric, NSCLC, and squamous cell carcinoma [22, 24, 53, 54, 55, 56]. Certain signaling mediators, such as mTORC1, TP73, and Beclin‐1, are crucial for the proper tumor suppressive activity of *DEPDC2* [22, 57, 58].

Previous studies have described the function of ncRNAs, particularly miRNAs, in regulating the expression of *DAPK2*. For instance, a study by Su et al. [59] revealed the upregulation of miR‐520h in breast cancer cell lines and tissues compared with normal cases. It was found that miR‐520h interferes with *DAPK2* expression post‐transcriptionally by directly binding to the 3′‐UTR of *DAPK2* mRNA, thereby protecting cancer cells from apoptosis [60]. Moreover, miR‐520h can inhibit paclitaxel‐induced apoptosis in breast cancer by suppressing the expression of *DAPK2* and caspases [59]. Hence, *DAPK2* could be considered as a crucial gene that triggers cell death and suppresses cell proliferation in breast cancer cells. Similarly, another study reported that *DAPK2* upregulation induces cell arrest and apoptosis in colorectal cancer. In their study a miR‐1285 binding site was identified in *DAPK2* mRNA. miR‐1285, an oncogenic miRNA, targets *DAPK2* mRNA and inhibits its expression in colorectal cancer. Therefore, inhibition of miR‐1285 activity can stimulate apoptosis and lead to cell cycle arrest [55]. In another study, c‐myc was shown to regulate miR‐106 expression in T24 cells. A significant decrease in *DAPK2* levels has been reported in bladder cancer cells because miR‐106 efficiently binds to the 3′‐UTR of *DAPK2*. Consequently, miR‐106 suppresses *DAPK2* transcription and inhibits apoptosis in bladder tumor cells [61]. Another study showed that lncRNA *MIAT* could function as an opposing endogenous RNA to increase *DAPK2* levels by sponging miR‐22‐3p, ultimately resulting in the apoptosis of cardiomyocytes, a critical process involved in the development of diabetic cardiomyopathy [62].

The contribution of lncRNAs to the progression and development of cancer has been established in numerous studies [63]. These molecular regulatory functions are based on their sequences and structures. These can form secondary and tertiary structures with different domains, with the potential to interact with proteins, chromatin, and other transcripts [64]. For instance, the nuclear lncRNA *HOTAIR* function has been well investigated, and it was found that it inhibits the transcription of *HoxD* cluster genes by binding to the PRC2 complex of the epigenetic mechanism to regulate histone modifications in trans. *HOTAIR* has been demonstrated to modify the chromatin state during tumor metastasis and is increased in metastatic breast carcinomas, leading to a modified distribution of PRC2 activity from embryonic fibroblasts to breast epithelial cells [65, 66].

*LOC101928988* is a type of lncRNA known as antisense lncRNA, which is located on the *DAPK2* gene region. Antisense lncRNAs can manage the transcription and expression of different genes and regulate various signaling pathways. LncRNAs perform these functions through cis or trans actions. In cis action, an lncRNA interacts with the promoter region with precise sequence complementarity, while in trans action, it interacts with defective sequence complementarity [67]. These lncRNAs were identified in both the nucleus and the cytoplasm. Compared with those present in the nucleus, antisense lncRNAs are more common in the cytoplasm. These cytoplasmic lncRNAs function as regulators of the mRNA degradation and protein synthesis processes [68, 69].

Nuclear lncRNAs regulate the transcriptional landscape by interacting with chromatin remodelers, like polycomb complex's members. These lncRNAs frequently serve this purpose in cancer [70]. Recently it was indicated that upregulation of lncRNA *PANDAR*, leading to the conduction of G1/S transition and promoting cell proliferation in breast cancer cells. Further experiments revealed that interaction of *PANDAR* with the Bmi1, a member of PRC1 complex, decrease the p16INK4A level [71, 72]. Also, it has been discovered that oncogenic lncRNA, *linc00511*, is overexpressed in ER^−^ breast cancer. This lncRNA inhibits cell death and accelerates the G1/S transition, leading to a worse patient prognosis. *Linc00511* recruits EZH2 to the *CDKN1B* promoter. Notably, EZH2 acts as the catalytic subunit of PRC2 complex [73, 74].

In another study, the nuclear lncRNA *EGOT1* was found at lower levels in breast cancer tissues. It is transcribed in an antisense direction from the ITPR1 gene's intronic regions. Its association with microtubule function suggests its possible influence on the response to paclitaxel, a chemotherapeutic drug. Further studies showed the direct binding ability of *EGOT1* to *IPTR1* pre‐mRNA, which led to the formation of double‐stranded RNA and regulation of *ITPR1* expression. This finding demonstrates that *EGOT1* plays a role both within and beyond the *ITPR1* gene, which enhances the efficiency of paclitaxel treatment in breast cancer cells [75].

The results of our study revealed that changes in the expression of *DAPK2* and *LOC101928988* could be correlated with the initiation and progression of breast cancer. It seems that *LOC101928988* has a regulatory effect on *DAPK2* expression. Complementary studies using RNAInter and GeneCards databases revealed that *LOC101928988* may interact with a number of TFs and RBP that have a target site on the promoter of *DAPK2*. Docking analysis showed that *LOC101928988* could interact with HNRNPL, CTCF, EZ2H, REST, and COREST. These factors contribute to epigenetic changes, especially DNA methylation. It seems that *LOC101928988* acts as a scaffold for these factors and plays a role in the assembly of the complex that mediates methylation of the *DAPK2* promoter. The abovementioned hypothesis was further underscored by the docking analysis results, which unveil distinct interaction positions for each element within *LOC101928988*. Crucially, the binding of each factor did not impede the subsequent binding of other factors, thereby fortifying the hypothesis. Confirmation of this hypothesis requires functional and supplementary studies. According to the results of the ROC curve analysis, *LOC101928988* has the potential to be further investigated as a diagnostic and prognostic panel. Further studies with larger statistical populations and functional tests, such as RIP, are recommended to validate the performance of *LOC101928988*. Although our analysis offers predictive insights into the interactions between *LOC101928988* and *DAPK2*, computational predictions necessitate empirical validation to confirm their biological significance. To resolve this, it is proposed to undertake more research, including co‐immunoprecipitation assays to verify direct contacts between *LOC101928988* and *DAPK2*, as well as luciferase reporter assays to evaluate the regulatory influence of *LOC101928988* on *DAPK2* promoter activity.

Moreover, the found predictive efficacy of 74% for *LOC101928988*'s target, *DAPK2*, prompts significant issues concerning its therapeutic relevance. Although our analysis offers significant insights into the regulatory link between these two substances, it is essential to recognize that the sample cohort was predominantly classified according to pathological phases rather than molecular subtypes. This methodological decision may restrict the generalizability of our results, given the heterogeneity and diverse genetic features associated with breast cancer. Subsequent research should focus on stratifying patient samples based on recognized molecular subtypes, including luminal A, luminal B, HER2‐enriched, and triple‐negative breast cancer. This approach enables us to clarify the exact settings in which *LOC101928988* may regulate *DAPK2* expression and potentially identify patient subgroups that could benefit most from focused therapy efforts employing this lncRNA. These investigations would not only clarify *LOC101928988*'s function in breast cancer biology but also augment its potential as a predictive biomarker in personalized medicine.

## 5 Conclusion

This study includes a well‐defined objective centered on understanding the regulatory relationship between *LOC101928988* and *DAPK2* in breast cancer, which addresses a critical gap in the current cancer research landscape. Our findings revealed that dysregulation of *DAPK2* and *LOC101928988* might play a role in the initiation and progression of breast cancer. Moreover, it was proposed that *LOC101928988* may be involved in regulating *DAPK2* gene expression through interaction with TFs. The use of paired tumoral and normal tissue samples from breast cancer patients allows for a robust comparison of gene expression in a relevant clinical setting, enhancing the ecological validity of the findings. However, the relatively small sample size may restrict the generalizability of the results. Further extensive functional validation of the mechanisms by which *LOC101928988* regulates *DAPK2* is required and, investigations aimed at employing *LOC101928988* as an integral part of a diagnostic and prognostic panel could be beneficial.

## Author Contributions

**Mohammadreza Saberiyan:** conception and design of the study, data analysis and writing the manuscript. **Nazila Ghasemi**, **Negar Jafari**, **Mahboubeh Sadeghi**, **Abbas Ghaderi:** acquisition of data, data analysis, and writing the manuscript. **Pegah Mousavi:** contributed to the study's conception and design as well as its writing. The final manuscript version was reviewed by all authors and approved.

## Conflicts of Interest

The authors declare no conflicts of interest.

## Data Availability

Upon request, the corresponding author will provide the data supporting the study's conclusions. The statistics are not accessible to the general public because of ethical and privacy concerns.
